# Prevalence of oral submucous fibrosis linking with Areca Nut usage among Indians

**DOI:** 10.6026/973206300200751

**Published:** 2024-07-31

**Authors:** Abhishek Jayaswal, Saurabh Goel, Kavita Verma, Srujal Jivrajani, Barkha Makhijani

**Affiliations:** 1 Department of Oral Medicine and Radiology, Pacific Dental College and Research Center, Udaipur, Rajasthan, India; 2 Department of Periodontics, Pacific Dental College and Research Center, Udaipur, India

**Keywords:** Areca nut, oral submucous fibrosis, Indians

## Abstract

The prevalence of oral submucous fibrosis amongst population of Southern Rajasthan is of interest to dentists. Hence, a cross sectional study had been conducted on 3548 patients from 18-60 years age group who visited the Department of the Oral Medicine and Radiology, Pacific Dental College and Research Centre, Bedla, Udaipur was completed. They were subjected to thorough case history related to quid habit in arecanut (*Areca catechu L.*) form and to diagnose OSMF clinically. 1645 processed form of arecanut users were identified. The prevalence of arecanut chewers in study population has been reported to be 46.36%. The prevalence of OSMF in study population has been reported to be 458 (12.9%). Exact component associated and the mechanism involved in the occurrence of OSMF is still not available in literature and research is going on regarding unveiling this mysterious lesion.

## Background:

Oral Submucous Fibrosis (OSMF) is a potentially malignant disorder (PMD) and crippling condition of oral mucosa [1]. It was first reported by Schwartz in 1952 [2] among five Indian females from Kenya and he designated the term '*Atropica idiopathica* mucosae oris' to this condition [3]. In 1953, Joshi described this condition as 'submucous fibrosis' [4]. Oral submucous fibrosis (OSMF) as described in 1966 by Pindborg and Sirsat is an insidious, precancerous, chronic disease that may affect the entire oral cavity and that sometimes extends to the pharynx. Although it is occasionally preceded by the formation of vesicles, OSMF is always associated with a sub-epithelial inflammatory reaction followed by fibro-elastic changes of the lamina propria, accompanied by epithelial atrophy. This process leads to stiffness of the oral mucosa, which results in trismus and inability to eat [5]. Warnakulasuriya *et al.* [6] defined a series of symptoms and subjective signs which are characteristic of OSMF to be employed as the clinical criteria required making a diagnosis of OSMF. The changes of OSMF are similar to those of systemic sclerosis (scleroderma) but are limited to oral tissues. OSMF is most commonly found in the age group of 20-40 years although it can occur in any decade. It is predominantly seen in people of South Asia and South-East Asia where consumption of arecanut or its flavoured formulations or as an ingredient in the betel quid is more prevalent. Variations in the prevalence figures are common between different studies, probably because of differences in the clinical criteria for diagnosis. The prevalence rate in India is about 0.2-0.5% [7]. The aetiology of OSMF is multifactorial but arecanut chewing is the main causative agent. Once initiated, OSMF is not amenable to reverse at any stage of the disease process even after cessation of the putative causative factor of arecanut chewing. The condition may remain either stationary or become severe, leaving an individual handicapped, both physically and psychologically [8]. It has been proven that there is a positive association between the incidence of leukoplakia and oral cancer with OSMF. The frequency of malignant change has been reported from 3% to 6% [9]. The possible precancerous nature of OSMF was first described by Paymaster, who observed the occurrence of squamous cell carcinoma in one third of his patients with OSMF. The incidence of malignant change in patients with OSMF reported to be ranging from 2 to 10% [10]. Few studies have been reported in literature related to the occurrence of OSMF with respect to the usage of processed forms of quid containing flavoring agents like menthols, cinnamon and preservatives like condiments and spices that are being added in processed packets% [11]. Therefore, it is of interest to assess the prevalence of OSMF amongst the population of Southern Rajasthan.

## Materials and Methods:

A cross sectional study had been conducted on 3548 patients from 18-60 years age group who visited the Department of the Oral Medicine and Radiology, Pacific Dental College and Research Centre, Bedla, Udaipur after obtaining ethical clearance from the ethical committee. An informed consent had been taken for all the patients. They were subjected to thorough case history related to quid habit in arecanut form and a detailed clinical examination of the oral cavity was carried out under artificial light. Patients who have discontinued the habit for two years or more, having other deleterious habits like tobacco, smoking, alcohol, drug addiction or other drugs, with restoration, metallic crowns, Any history of grafts placement and treatment undertaken for any oral mucosal lesions had been excluded from the study. OSMF clinically was evaluated using the modified criteria given by W.H.O [12]. Habit counseling was done followed by periodic recall.

## Results and Discussion:

The present study was conducted screening 3548 subjects that had reported to the dental college during a period of 1 year and 1645 processed form of arecanut users were identified. The prevalence of arecanut chewers in study population has been reported to be 46.36%. The prevalence of OSMF in study population has been reported to be 458 (12.9%). Variability in the results has been noted in accordance with the prevalence reported by Nasir Jamal Baig *et al.* in 2012 (13%) [13], Kumar Nigam *et al.* in 2014 (6.3%) [14] .Variability in the results has been noted according to the region wise variations in the usage of quid. Quid involving arecanut is normally harvested as unripe (yellow-green) or ripe (orange/red) fruit from the tropical palm, Areca catechu. The Areca fruits may be sun dried for several weeks, fibrous shells removed and the hard, dry nuts, commonly called supari in India, are ready for use in raw form. Alternatively, the ripe arecanut are boiled for several hours in an aqueous solution containing the bark of the plant Eugenia jambolana, jaggery or brown sugar, and various edible oils, to 'cure' it [15]. Areca nut is made up of alkaloid and flavonoid components. Four alkaloids namely arecoline, arecaidine, guvacine, and guvacoline have been identified in areca nut, of which arecoline is the most potent agent and plays a major role in the pathogenesis of OSF by causing an abnormal increase in collagen production. The flavonoid components like tannins and catechins have been found to have some direct influence on collagen metabolism [16] Basic mechanisms involved in the pathogenesis of OSF can be divided into four steps: Occurrence of the chronic inflammation at the site of betel quid placement, Increased collagen synthesis, collagen cross-linking, decreased collagen degradation leading to occurrence of the chronic inflammation at the site of betel quid placement [16]. Studies have also found that chewing dried areca nuts are more pathogenic and carcinogenic than chewing fresh areca nuts, and the extremely harmful ingredients in areca nuts play a vital role in the occurrence of oral mucosal diseases and oral cancer [17]. Exact component associated and the mechanism involved in the occurrence of OSMF is still not available in literature and research is going on regarding unveiling this mysterious lesion.

## Conclusion:

The occurrence of OSMF with respect to the usage of processed forms of arecanut quid containing flavouring agents like menthols, cinnamon and preservatives like condiments and spices being added in processed packets should be studied to ascertain if they are associated with chewing arecanut.

## Financial support:

NIL
